# The effectiveness of the ketogenic diet in treating refractory epilepsy: A study of different seizure forms

**DOI:** 10.1097/MD.0000000000044557

**Published:** 2025-09-26

**Authors:** Meng Wang, Hongwei Zhang, Pingping Tian, Wandong Hu, Yehong Chen

**Affiliations:** a Neurology Department, Children’s Hospital Affiliated to Shandong University, Jinan, China; b Neurology Department, Jinan Children’s Hospital, Jinan, China; c Department of Electrophysiology, Epilepsy Center, Children’s Hospital Affiliated to Shandong University, Jinan, China; d Department of Electrophysiology, Epilepsy Center, Jinan Children’s Hospital, Jinan, China.

**Keywords:** different seizure form, ketogenic diet, refractory epilepsy, therapeutic effects

## Abstract

The objective of this study was to compare the efficacy of the ketogenic diet (KD) in patients with refractory epilepsy with different seizure forms and to further evaluate the correlation between seizure types and the efficacy of KD. In this prospective observational cohort study, consecutive children with refractory epilepsy (N = 72) treated with KD were recruited for follow-up. The children were divided into 2 groups: a nongeneralized seizure group and a full seizure group. Monthly seizure frequencies and seizure reduction rate after KDT 3 months and 6 months were compared between 2 groups. Follow-up results at 3 and 6 months showed a decrease in seizure frequency in both the nonprolific and full seizure groups, and the differences in seizure frequency from baseline were statistically significant in both the nonprolific and full seizure groups. After 3 months of KD treatment, the seizure frequency in the nongeneralized seizure group was notably lower than that in the full seizure group. However, after 6 months of KD treatment, there was no statistically significant difference between the seizure frequency of patients in the nongeneralized seizure group and those in the full seizure group. Further findings indicated a greater proportion of seizure reduction in the nongeneralized seizure group than in the full seizure group after 3 months of KD treatment. Statistically significant differences exist between the 2 groups (*P*＜.05). At the 6 months mark of KD treatment, no statistically significant disparities were observed in the seizure. Finally, this paper further proves the above point by comparing the number of patients who responded to KD through per-protocol population (PP) analysis and intention-to-treat analysis.

## 1. Introduction

Epilepsy is a prevalent neurological disorder with a wide range of etiologies that, manifest as recurrent seizures due to aberrant electrical transmission within the brain.^[1]^ Epidemiological estimates suggest that 5 to 1.7 million people worldwide are affected by epilepsy.^[2]^ According to the International League Against Epilepsy (ILAE), refractory epilepsy (RE) is a condition in which a patient exhibits seizures that remain unresponsive to a minimum of 2 anti-seizure medications (ASMs), whether in monotherapy or in combination therapy.^[3]^ Research has shown that 20% to 30% of pediatric patients with epilepsy who receive effective ASMs subsequently develop RE and require alternative treatment options.^[4]^

The ketogenic diet (KD) is a nutritional plan developed in the United States more than 90 years ago. It is characterized by elevated levels of fat, low levels of carbohydrates, and protein restriction. Since its development, KD has gained global popularity and has been adopted by individuals seeking health benefits.^[5]^ Mounting evidence suggests that the KD is a treatment for RE, and its effectiveness, safety, and tolerability in treating drug-resistant epilepsy has been demonstrated in children.^[6]^ KD therapies offer distinct benefits in the treatment of RE compared with ASMs. The efficacy of KD treatment in patients with drug-resistant epilepsy has been demonstrated by the fact that 15%–27.6% of patients have achieved seizure-free status.^[7]^ Previous research has also shown that dietary treatment has the potential to reduce the overall ASM use.^[8]^

Existing research indicates that KD is generally considered to be a relatively safe intervention for the majority of pediatric patients with epilepsy.^[9–11]^ However, the therapeutic efficacy of KD may vary in children with refractory epilepsy, which may be attributable to different seizure forms. Consequently, the present study was designed to compare the therapeutic efficacy of KD in patients with RE with different seizure forms and to further evaluate the correlation between seizure types and the efficacy of KD.

## 2. Materials and methods

### 2.1. Study design

A prospective cohort design was used to follow 72 children with refractory epilepsy treated with KD over a 6-month period, including outpatient hospitalization.

### 2.2. Settings

This study consecutively recruited children with refractory epilepsy treated with KD who attended the outpatient clinics and wards of the Epilepsy Center of the Affiliated Children’s Hospital of Shandong University between January 2022 and June 2024. The study was approved by the Ethics Committee of the Affiliated Children’s Hospital of Shandong University prior to initiation (Ethical Approval No. SDFE-IRB/*P*-2022054). All family members agreed to participate in this study and signed an informed consent form.

### 2.3. Participants

#### 2.3.1. *The inclusion criteria was as follows*

Meets the 2010 International League Against Epilepsy (ILAE) criteria for refractory epilepsy, Age ≤ 18 years, no previous treatment with KD, KD was initiated in children who had previously provided informed consent.

#### 2.3.2. *Exclusion criteria*

Unsuitable for KD or contraindications to KD therapy; subjects with known metabolic diseases or severe systemic illnesses; poor cooperation of the child or the child’s family for various reasons.

#### 2.3.3. Groups

All the subjects included in this study were divided into 2 groups based on how their seizures looked and their EEG results showed. The first group had generalized seizures, and the second group had nongeneralized seizures. All children were given the “classic” KD program (CKD), meaning that the fat, protein, and carbohydrate portions were 4:1:1.

### 2.4. KD Program

#### 2.4.1. Starting the KD

KD was initiated in children at the Epilepsy Center of the Affiliated Children’s Hospital of Shandong Province, where metabolic screening, including tests for blood ammonia, lactate, serum cholesterol, triglycerides, and urinary organic acid levels, was performed to rule out contraindications to KD prior to the initiation of treatment. Non-fasting progressive KD was employed as the initial protocol following screening, and a registered dietitian calculated each patient’s energy requirements based on age, weight, height, daily activities, and dietary history. The KD ratio was initiated at 1:1 and transitioned to 3:1, with incremental adjustments to ensure adequate protein intake and meet the growth and development requirements of children. The ratio of the diet was subsequently modified between 2:1 and 4:1, with the objective of maintaining blood ketones at 3 to 5 mmol/L and blood glucose at 4 to 5 mmol/L, while ensuring seizure control and maximizing quality of life. The final individualized KD protocol was developed with the primary objective of optimizing quality of life. At the time of initiation, the original anti-seizure medication was maintained. Blood ketones and blood glucose levels were monitored daily during hospitalization. No serious adverse effects were observed in patients with stable blood ketone levels. Following discharge, the KD protocol was continued with regular follow-up, during which blood glucose and ketone levels were tested weekly.

#### 2.4.2. Follow-up and outcome assessment indicators

The occurrence of seizures was meticulously documented by a designated guardian, who recorded the frequency of these events in a designated seizure diary. Following their inclusion in the study, all patients were followed-up through outpatient clinics and by telephone. Parents were instructed to maintain a daily ketogenic diary and to visit the hospital outpatient clinic on the 3rd and 6th months for follow-up assessment and prompt symptomatic management of any adverse reactions. The secondary outcome was to determine the effectiveness of KD by calculating the degree of seizure reduction after 3 and 6 months of KD treatment. The seizure reduction rate was calculated as follows: first, the number of seizures at the 28-day baseline period (a) was determined, and then the number of seizures at 3 or 6 months of follow-up (b) was recorded. Subsequently, the formula used to calculate the seizure reduction rate was expressed as [(a-b)/a] × 100.

### 2.5. Statistical analyses

In this study, the Mann–Whitney *U* test was employed to analyze the count data (e.g. age, number, frequency of seizures before and after the intervention), and the chi-square test (frequency > 5) or Fisher test (frequency < 5) was used to compare the demographic data (i.e. sex) of the 2 groups of patients. The intention-to-treat (ITT) population comprised of all patients who met the established eligibility criteria and were assigned to receive an intervention. In instances where patients could not be contacted at 3 or 6 months following the intervention, the mean daily seizures recorded over the final 4 weeks of the most recent contact with the patient were used for the primary endpoint calculation. The per-protocol population (PP) was defined as the subset of patients who did receive the full allocated treatment up to 3 or 6 months. The resulting p-values will be interpreted in a descriptive manner, i.e. no formal hypothesis testing will be performed.

## 3. Results

### 3.1. Patient enrollment and diet adherence rate

**Figure 1.**
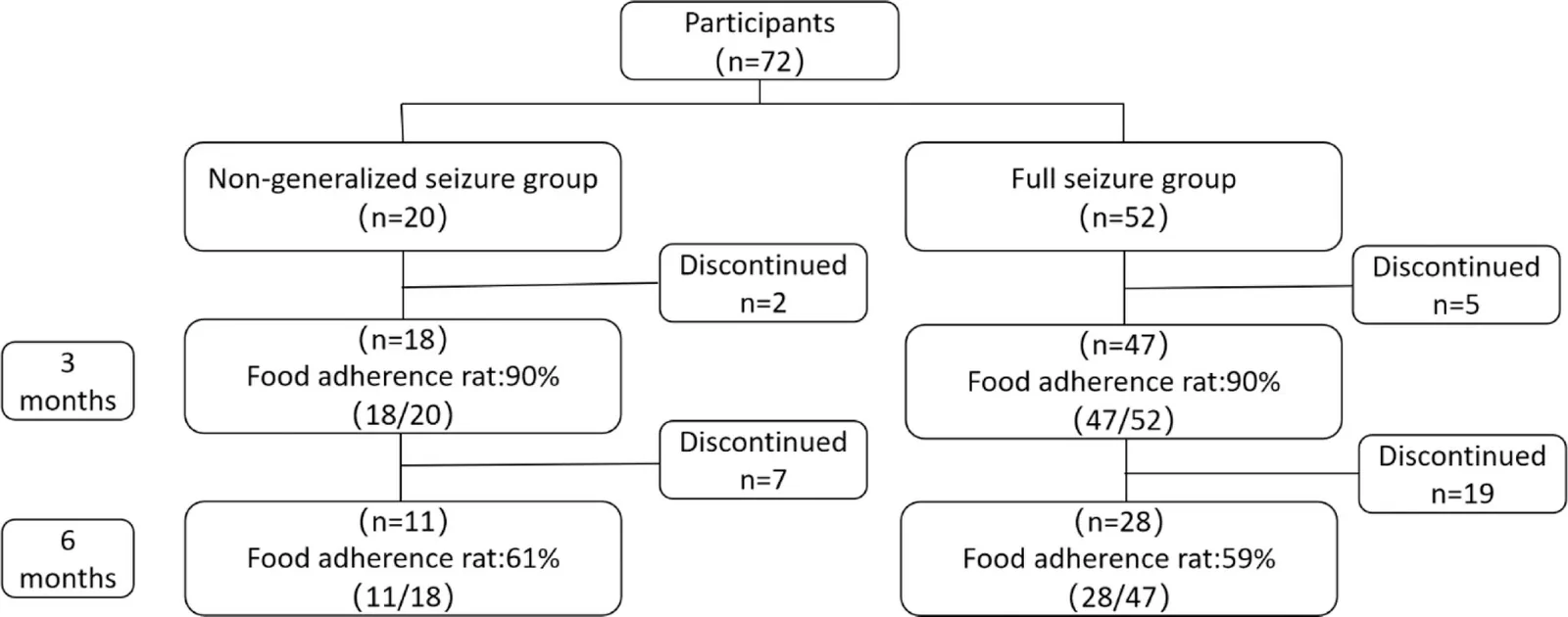
Illustrative flow chart of study protocol.

Figure 1 demonstrates patient recruitment and diet adherence rate throughout the 6 months of KD follow-up. The present study was conducted on a cohort of 72 children diagnosed with RE, who were treated with KD. The sample population included 20 children in the nongeneralized seizure group and 52 in the generalized seizure group. Of the 20 patients in the nongeneralized seizure group, 2 withdrew from the KD regimen before 3 months of follow-up. The 3-month KD adherence rate was 70% (18/20 patients). Seven patients withdrew from the dietary program before the completion of 6 months of follow-up, leaving 11 patients who remained on the dietary program. The 6-month dietary adherence rate was 55% (11/20 patients). Of the 52 patients in the full seizure group, 5 withdrew from the KD regimen before 3 months of follow-up. The 3-month KD adherence rate was 90.4% (47/52 patients). 23 patients withdrew from the dietary program prior to the completion of 6 months of follow-up, leaving 28 patients who remained on the dietary program. The 6-month dietary adherence rate was 53.8% (28/52 patients).

### 3.2. Patients’ demographics

As shown in Table 1, 72 patients on the KD were divided into 2 groups with no significant differences in sex, age to start of KD, at to start KD minus age at first seizure, ASMs at KD, family history, birth history and hereditary history.

**Table 1:** Demographic data of study subjects.

Characteristic	Overall, N = 72	Nongeneralized seizure group (N = 20)	Full seizure group (N = 52)	*χ*^2^/U	*P*
Gender n (%)					
Male	44 (61%)	12 (60%)	32 (62%)	0	1.000
Female	28 (39%)	7 (40%)	20 (38%)		
Age to start KD	21 (12, 36)	21 (11, 44)	21 (12, 36)	411	.866
Age to start KD minus age at first seizure	9 (4, 24)	13 (7, 24)	7 (4, 28)	490	.330
ASMs at KD (Median)	3.0 (2.0, 5.0)	3.0 (2.0, 9.0)	3.0 (2.0, 4.0)	440	.806
Family history					
No	61 (85%)	17 (85%)	44 (85%)		1.000
Yes	11 (15%)	3 (15%)	8 (15%)		
Birth history					
No	59 (82%)	18 (89%)	41 (79%)		.485
Yes	13 (18%)	2 (10%)	11 (21%)		
Hereditary history					
No	47 (65%)	12 (60%)	35 (67%)	0.057	.811
Yes	25 (35%)	8 (40%)	17 (33%)		

### 3.3. KD effectiveness

As shown in Table 2, the mean monthly seizure frequency at the start of the study KD was 147.9 ± 182.5 seizures/month in the nongeneralized seizure group and 142.5 ± 143.1 seizures/month in the full seizure group. There was no significant difference in the baseline seizure frequency between the 2 groups (*P* > .05). After 3 months of KD treatment, the monthly mean number of seizures decreased to 11.7 ± 19.7 seizures/month in the nongeneralized seizure group and 64.11 ± 93.7 seizures/month in the full seizure group. The frequency of seizures was significantly lower in the nongeneralized seizure group than in the full seizure group (*P* < .05). After 6 months of KD treatment, the monthly mean number of seizures decreased to 68.8 ± 139.2 seizures/month in the nongeneralized seizure group and 28.09 ± 45.0 seizures/month in the full seizure group. The seizure frequency in children in the nongeneralized seizure group was not significantly different from that in the full seizure group (*P* > .05).

**Table 2:** Three months and 6 months KDT outcome for study subjects.

	Baseline	P0	3M	P1	6M	P2
Seizure frequency (seizures/month)						
Full seizure group	142.5 ± 143.1	0.55	64.11 ± 93.7	0.04	68.8 ± 139.2	0.83
Nongeneralized seizure group	147.9 ± 182.5		11.7 ± 19.7		28.1 ± 45.0	
**Responder rate**			**3M**	**p1**	**6M**	**P2**
PP analysis						
Full seizure group (n = 52)	–	–	51% (24/47)	0.02	59% (17/29)	0.49
Nongeneralized seizure group (n = 20)	–	–	83% (15/18)		73% (8/11)	
ITT analysis						
Full seizure group (n = 52)	–	–	46% (24/52)	0.04	32% (17/52)	0.69
Nongeneralized seizure group (n = 20)	–	–	75% (15/20)		40% (8/20)	

ITT = intention-to-treat, KDT = ketogenic diet therapy, P0 = full seizure group vs nongeneralized seizure group at baseline, P1 = full seizure group vs nongeneralized seizure group at 3 months, P2 = full seizure group vs nongeneralized seizure group at 6 months, PP = per-protocol population.

**Figure 2.**
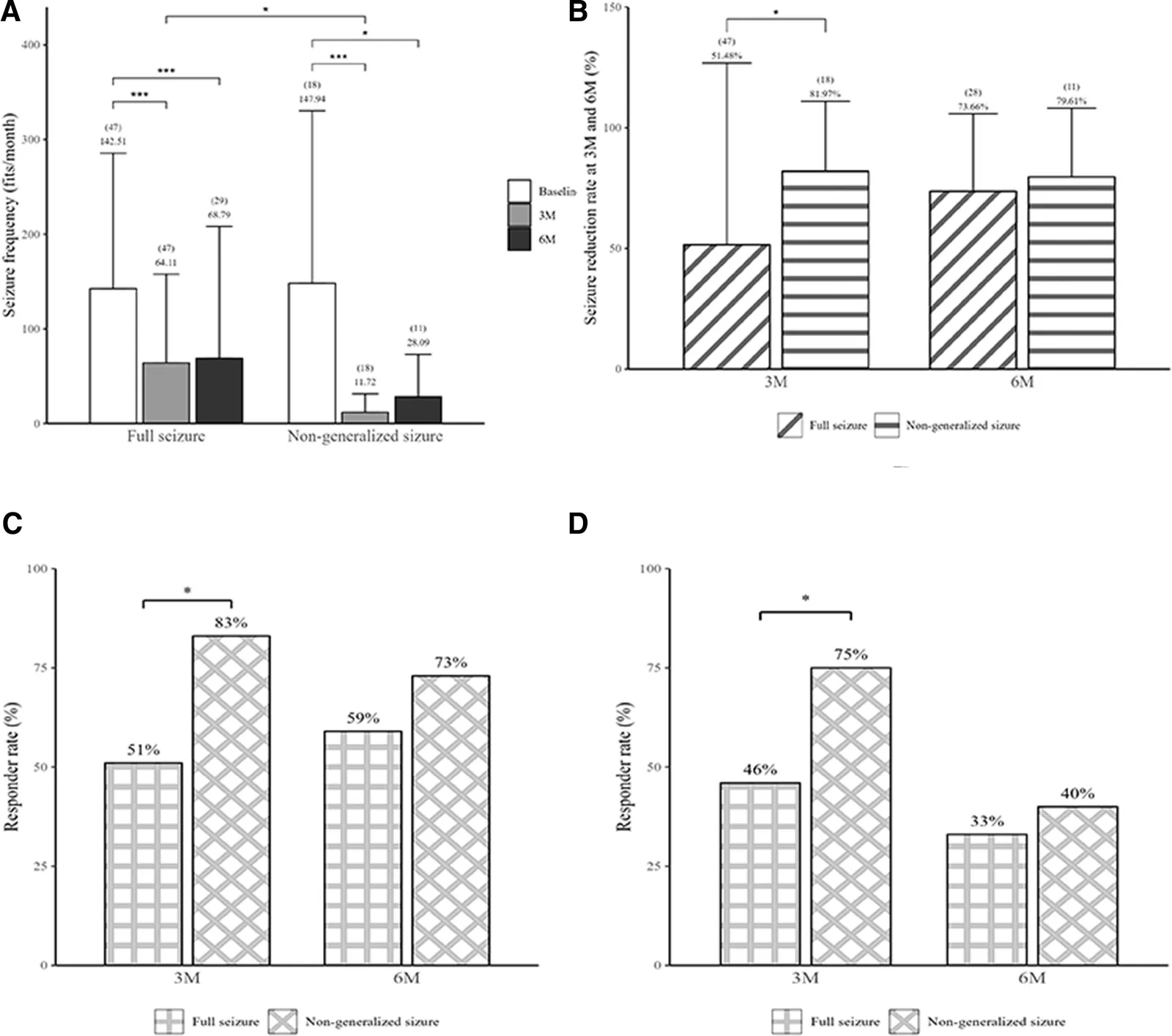
Primary outcome and secondary outcome of KD. 2A. Seizure frequency in patients in the full seizure group or the nongeneralized seizure group t baseline, after 3 mo of KD, and after 6 mo of KD.2B. Seizure reduction rate in patients with full seizure group or a nongeneralized seizure group t baseline, after 3 mo of KD, and after 6 mo of KD.2C. PP analysis: response rate of children in the full seizure group or the nongeneralized seizure group at baseline, after 3 mo of KD, and after 6 mo of KD. 2D. ITT analysis: response rate of children in the full seizure group or the nongeneralized seizure group t baseline, after 3 months of KD, and after 6 months of KD. *P < .05; **P < .01; ***P < .001. ITT = intention-to-treat, KD = ketogenic diet, PP = per-protocol population.

As shown in Figure 2A, the nongeneralized seizure group had a significantly lower seizure frequency at 3 and 6 months of KD compared to the baseline seizure frequency when receiving either 3 or 6 months of fully allocated treatment (3 months vs baseline, *P* < .001; 6 months vs baseline, *P* < .001). Patients in the full seizure group who received KD therapy for up to 3 or 6 months had significantly lower seizure frequency at 3 and 6 months of KD treatment than at baseline (3 months vs baseline, *P* < .001; 6 months vs baseline, *P* < .05). After 3 months of KD treatment, the seizure frequency of patients in the nongeneralized seizure group was significantly lower than that of patients in the full seizure group (*P* < .05). However, after 6 months of KD treatment, the difference between the seizure frequency of patients in the nongeneralized seizure group and that of patients in the full seizure group was not statistically significant (*P* > .05). The findings indicated that following a 3-month period of KD treatment, seizure frequency notably diminished, particularly among patients in the nongeneralized seizure category. However, this disparity in seizure frequency gradually declines over time. After a 6-month duration of KD treatment, seizure frequency in the full seizure group did not demonstrate a statistically significant difference compared to that in the nongeneralized seizure group.

As demonstrated in Figure 2B, at the 3-month mark of KD treatment, the seizure reduction rate among children in the nongeneralized seizure group surpassed that of patients in the full seizure group (*P* < .05). At the 6-month mark of KD treatment, no substantial disparities in seizure reduction rates were observed between children in the nongeneralized seizure group and those in the full seizure group. This is another aspect that demonstrates the regularity of the effects of seizure form on the therapeutic efficacy of KD treatment.

We also compared the number of patients who responded to KD by calculating the “response rate.” As shown in Table 2 and Figures 2B and C, after 3 months of KD intervention, the response rate in the full seizure group was significantly higher than that in the nongeneralized seizure group in the PP (*P* = .02) and ITT (*P* = .04). However, after 6 months of KDT intervention, the response rate in the nongeneralized seizure group was similar to that in the full seizure group in the PP analysis (*P* = .49) and ITT analysis (*P* = .69).

### 3.4. Analysis of adverse reactions and shedding in the ketogenic group

KDT treatment resulted in gastrointestinal adverse reactions in 11 cases (15.3%), including vomiting (6 cases) and constipation (7 cases). Two (2.78%) children experienced sleepiness. These reactions occurred in the early stages of KD treatment, and 6 cases improved after adjusting the proportion of diet and medication, allowing them to continue KD treatment. There were 3 (4.2%) urinary adverse reactions, 1 hyperuricemia and 2 urinary stones, all of which were terminated during the course of treatment. One child developed significant weight loss and dyslipidemia (1.3%) at the 4th month after oral administration. 38 (28.9%) patients were dislodged at 6 months due to loss of visits, dietary side-effects, and inability to adhere to dietary habits.

## 4. Discussion

Since the 1920s, the KD has been considered a possible treatment for epilepsy. However, it has been put on hold for a significant period due to the extensive development and use of antiepileptic medications (ASMs) in clinical practice. In the past 2 decades, subsequent to continuous clinical research and basic experiments confirming the efficacy of KD in treating epilepsy (RE), KD has gained recognition as a treatment for RE.^[12–14]^ Despite the long-standing application of dietary therapy for RE, the precise mechanism by which it exerts its effects remains debate.^[15–18]^ KD has been observed to replicate the metabolic shifts that transpire during periods of starvation by modulating the proportion of fat and carbohydrates in one’s diet, thereby affecting the brain’s energy supply and seizure management. It has been increasingly acknowledged that KD exerts a multifaceted effect by modulating the levels of neurotransmitters, affecting the energy status of cells, increasing the concentration of ketone bodies and polyunsaturated fatty acids, altering the inflammatory response, regulating cellular autophagy, apoptotic processes, and influencing the gut microbiota.^[16,19]^ The current state of research on contraindications to KD is insufficient. Therefore, it is recommended that a patient’s health status be evaluated each time. Available studies have shown that KD is relatively safe in most patients^.[20–22]^ The utilization of KD has undergone gradual expansion over time. With regard to the question of the initiation, Bergqvist et al.^[23]^ found that gradual initiation of the KD resulted in greater seizure reduction and fewer adverse events than fasting initiation. In the present study, KD was initiated gradually to reduce seizures and adverse events. The present study investigated the impact of the seizure form on the efficacy of KD in treating refractory epilepsy.

As previously stated, KDT is a prevalent treatment option for refractory epilepsy, with a documented efficacy rate ranging from 35% to 85% in patients with drug-resistant epilepsy.^[7,15,24]^ Another study by de Kinderen et al found a higher percentage of 50% reduction in seizures in the KD group compared to the no dietary intervention group.^[25]^ Lambrechts et al.^[26]^ found that at 4 months, children with > 90% and > 50% seizure reduction in the KD group were significantly more likely to have seizures than those in the no dietary intervention group. Similar findings have been reported by Neal et al.^[27]^ The findings of these studies indicate that a KD serves as a means to reduce the frequency and severity of seizures. Our results in the whole cohort study showed a response rate of 60% (39/65) after 3 months of KDT treatment and 82% (23/28) after 6 months of treatment, which is in line with previous studies and suggests that there is an important role for KDT in refractory epilepsy.

The influence of seizure type on KD for refractory epilepsy has often been discussed in previous studies^[28]^, Existing which have shown that partial-type seizures are the least effective for KD for refractory epilepsy,^[8,29]^ and that patients with generalized epilepsy or younger patients respond better to treatment^[16,20,30]^. The results of the study showed that KD was the most effective in reducing nonfocal seizures, while KD for focal seizures was the least effective treatment, with seizure reductions of only 42.9% and ≤ 50% in 57.1% of patients. However, if only patients with a known etiology were considered, focal seizures were treated effectively with a KD in 5 out of 6 cases (83.33%). In addition, KD treatment was ineffective in 60% (6/10) of patients with generalized tonic-clonic seizures when presented alone. However, when they were presented in conjunction with focal seizures, they were effective in 52.9% of the cases.^[8]^ Consequently, these findings have prompted physicians in clinical practice to exclude children who present solely with partial seizures, thereby narrowing the therapeutic scope of KD for treatment-refractory epilepsy. However, other studies have shown that the type of seizure dose not have a significant effect on the outcome of KD treatment.^[31,32]^ A study of treatment outcomes in 147 patients with refractory epilepsy revealed no significant differences in the treatment of KD according to seizure type.^[32]^ The results of this study showed were that after 3 months of KD treatment, the frequency of seizures was significantly reduced, especially in the patients in the nongeneralized seizure group (*P* < .05); however, this difference gradually decreased with time. However, after 6 months of KD treatment, the frequency of seizures in the full seizure group was not significantly different from that in the nongeneralized seizure group.

Follow-up of children with RE treated with KD showed a decrease in adherence over time and an increase in the rate of loss to follow-up in children with different seizure types. Adherence rates in the nongeneralized seizure group (n = 20) were 70% at 3 months and 55% at 6 months; in the full seizure group (n = 52), 90.4% at 3 months and 53.8% at 6 months, suggesting that the risk of dropping out between 3 and 6 months was higher. The 10% dropout within 3 months in the nongeneralized seizure group was slightly higher than the 9.6% in the full seizure group; the cumulative dropout rate at 6 months was close (45% vs 46.2%), which may be related to clinical characteristics associated with seizure type and adaptive capacity. Loss to follow-up may introduce selection bias. ITT and PP analyses showed that at 3 months, the response rate in the full seizure group was significantly higher than that in the nongeneralized seizure group (PP: *P* = .02; ITT: *P* = .04), which echoed the difference in adherence rates, and that there was no difference in the response rate between the 2 groups at 6 months (PP: *P* = .49; ITT: *P* = .69), either because of the similarity in adherence rates or because the KD efficacy rates were different between seizure types and the KD efficacy rates were similar at 6 months (45% vs 46.2%). Differences in KD efficacy between seizure types may be attenuated by equalization of the distribution of lost visits. The 6-month adherence rate in this study (53.8%–55%) is consistent with the literature and confirms long-term adherence.^[33]^ Subsequent studies can be optimized in 3 ways: incorporating the assessment of predictors of adherence and reducing baseline imbalance through stratified randomization; implementing targeted interventions, such as providing professional guidance and personalized dietary adjustments for high-risk groups; and continuing to use the dual analysis strategy of ITT and PP, combined with multiple interpolation to deal with the missing data in order to analyze the efficacy of KD in a more robust manner.

As demonstrated in previous studies, the administration of KD in the treatment of RE inevitably produces certain adverse effects. The most common adverse effects were gastrointestinal symptoms, including vomiting and constipation. Other adverse effects include somnolence, weight loss, dyslipidemia, metabolic acidosis, renal stones, hypoglycemia, and recurrent pneumonia. Constipation, somnolence, and anorexia are more common in the first few days of the diet and do not lead to dietary disruption.^[5,34]^ Several reports have suggested that long-term use of KD in children may result in growth retardation. However, the validity of these claims remains contentious and further research is required to substantiate these findings. In the early stages of treatment, patients may encounter challenges in adapting to a modified diet owing to the abrupt nature of the change. This can manifest as a reluctance to consume food or a decrease in dietary intake, leading to short-term hypoglycemia, lethargy, and metabolic acidosis. However, these adverse effects can be mitigated in the early stages of treatment and do not typically result in cessation of KD therapy.^[35]^ Several adverse effects were observed in the present study. During the initial 3-month period, 11 cases (15.3%) of gastrointestinal adverse reactions were documented, with 2 children experiencing somnolence (2.78%). Following dietary adjustments and treatments, only 4 children discontinued the KD, while the remainder were able to resume a normal diet. With the prolongation of KD, patients developed urinary adverse effects in 3 cases (4.2%) of which 2 had urinary stones and 1 had hyperuricemia. With the prolongation of KD, 3 (4.2%) children with KD developed adverse urinary effects, 2 of which were urinary stones and 1 with hyperuricemia. Kidney stones have been reported to occur in approximately 2.2% to 6.7% of patients treated for KD, and supplementation with potassium citrate and increased fluid intake may reduce stone size, with very few requiring surgical lithotripsy as a result. Mild hyperlipidemia is usually untreated, and severe hyperlipidemia can be controlled by lowering the ketogenic ratio and/or amount of saturated fat in the diet.^[36]^ In the present study, dietary treatment was terminated in all patients. One child with hyperuricemia normalized within 1 month of termination of ketogenic treatment in both cases, and in 2 cases, urinary stones were reduced in size and disappeared after treatment with oral herbal tonics.

## 5. Conclusion

Based on the data obtained, it can be concluded that KD is a safe and effective treatment option for refractory epilepsy. The effect of the seizure form on the efficacy of KD in the treatment of refractory epilepsy is related to time. In the short term, it is possible to see that patients with epilepsy in the nongeneralized seizure group outperform those in the full seizure group, but this difference narrows over time, and eventually no significant difference is seen. Therefore, when treating refractory epilepsy in KD, we cannot single-handedly rule out a certain seizure form of refractory epilepsy, and the decision should be made by comprehensively evaluating the patient’s condition. Additionally, considering the effectiveness and mild side-effects of KD, it seems reasonable to recommend this form of treatment in all refractory patients. However, our study has some limitations such as small sample size and single-center regression study design; therefore, a further large sample size and multicenter prospective studies are necessary.

## Author contributions

**Conceptualization:** Meng Wang, Yehong Chen.

**Data curation:** Meng Wang, Yehong Chen.

**Formal analysis:** Meng Wang, Pingping Tian, Yehong Chen.

**Funding acquisition:** Meng Wang, Yehong Chen.

**Investigation:** Meng Wang, Yehong Chen.

**Methodology:** Meng Wang, Yehong Chen.

**Project administration:** Meng Wang, Hongwei Zhang, Pingping Tian, Yehong Chen.

**Resources:** Meng Wang, Pingping Tian, Yehong Chen.

**Software:** Yehong Chen.

**Supervision:** Hongwei Zhang, Wandong Hu, Yehong Chen.

**Validation:** Meng Wang, Yehong Chen.

**Visualization:** Yehong Chen.

**Writing – original draft:** Meng Wang, Yehong Chen.

**Writing – review & editing:** Wandong Hu, Yehong Chen.
